# Retrospective Grid Cells in the Medial Entorhinal Cortex Encode Past Paths During Spatial Navigation

**DOI:** 10.1002/advs.77884

**Published:** 2026-09-27

**Authors:** Ruojin Liu, Xin Yuan, Zilong He, Hong Jin, Jiayi Tian, Cong Chen, Yuanjing Liu, Xiang Zhang, Shidan Wen, Emilio Kropff, Chenglin Miao

**Affiliations:** ^1^ State Key Laboratory of Membrane Biology School of Life Science Peking University Beijing China; ^2^ PKU‐IDG/McGovern Institute For Brain Research Peking University Beijing China; ^3^ Peking‐Tsinghua Center For Life Sciences Beijing China; ^4^ Peking University Chengdu Academy for Advanced Interdisciplinary Biotechnologies Chengdu China; ^5^ Institute of Information Engineering Chinese Academy of Sciences Beijing China; ^6^ Leloir Institute CONICET Buenos Aires Argentina; ^7^ Chinese Institute For Brain Research Beijing China

**Keywords:** computer science, encode, entorhinal cortex, grid, medial entorhinal cortex, neuroscience, sensory system, spatial memory

## Abstract

Neurons dynamically switch between prospective and retrospective coding depending on task demands. Prospective coding optimizes future actions, while retrospective coding helps learn from past experiences. The grid cells in the medial entorhinal cortex (MEC) play a key role in spatial navigation, with periodic firing fields to encode current position. While their role in prospective coding has been reported recently, whether and how grid cells exhibit retrospective coding (encoding past paths) remains unclear. Here, we combined large‐scale two‐photon imaging in freely moving mice with well‐established analytical approaches to identify and characterize retrospective grid cells in the MEC. We identified the retrospective grid cells, which encoded path history, showing maximal gridness score at backward‐shifted positions (∼15 cm), independent of calcium transient kinetics. Retrospective grid coding showed reduced spatial precision in darkness and was disrupted during assisted movement, indicating that its stable expression depends on sensory and behavioral conditions. In simulations, incorporating retrospective information into a path‐integration model reduced accumulated position error and improved model performance across varying movement speeds. These findings support a dual‐coding framework where retrospective and prospective cells jointly optimize navigation accuracy.

## Graphical abstract

Retrospective grid cells are identified in the medial entorhinal cortex, with spatial firing patterns that become most grid‐like when activity is projected onto recently traversed locations. Their coding loses spatial precision in darkness and is disrupted during assisted movement, indicating that entorhinal representations incorporate recent path history and vary across sensory and behavioral conditions.

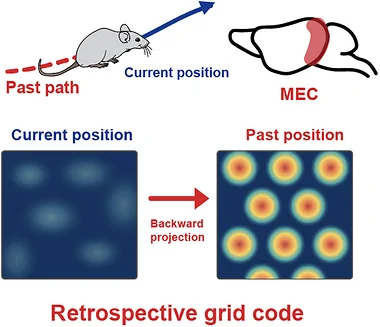

## 1 Introduction

The brain flexibly employs prospective and retrospective coding strategies to guide behavior—anticipating future states or reflecting on past experiences to optimize decision‐making. Prospective coding, a framework rooted in Bayesian inference, posits that the brain generates top‐down expectations to minimize prediction errors [1, 2]. For example, neuroimaging studies show that the prefrontal cortex and hippocampus actively predict reward outcomes during learning tasks [3, 4, 5], while sensory cortices suppress expected stimuli (e.g., learned visual cues, repeated sounds) to prioritize novel inputs [6, 7]. Conversely, retrospective coding is evident in memory consolidation: the hippocampus replays past sequences during rest to reinforce learning [8], and dopamine neurons signal past reward discrepancies to update future choices [9, 10, 11]. Dynamic shifts between these strategies are context‐dependent; during uncertainty, the brain weights retrospective evidence more heavily [12, 13], whereas familiar tasks rely on prospective schemas [6, 14, 15]. This duality underscores the brain's efficiency in balancing anticipation aptation.

The medial entorhinal cortex (MEC) serves as the brain's primary metric spatial encoder, where grid cells generate hexagonal firing patterns to tessellate the environment into a navigational coordinate system [16, 17, 18, 19, 20, 21, 22, 23, 24]. While these cells are classically characterized by their capacity for path integration—continuously updating position estimates through self‐motion cues [25]—recent theoretical and experimental work suggests the MEC network may encode both future (prospective) and past (retrospective) spatial information [26, 27, 28, 29, 30, 31, 32, 33, 34]. This dual temporal representation could provide a mechanistic basis for error correction in navigation, reconciling accumulating path integration drift with sensory landmarks [35, 36, 37]. However, whether grid cells explicitly exhibit retrospective coding, and how such coding depends on external (visual) versus internal (self‐motion) cues, remains unresolved, a critical gap given its implications for models of spatial memory and computational frameworks [38, 39]. Existing research has primarily focused on prospective coding in grid cells. Previous studies demonstrate that grid fields can predict imminent trajectories [32], with theta‐phase precession enabling temporal compression of upcoming paths [40]. Conversely, retrospective coding—the encoding of recently traversed locations—has received limited attention, despite its potential role in error correction. Theoretical models propose that such retrospective activity could serve as a “lookback” mechanism to align path integration with boundary cues [39], but direct empirical evidence is lacking. With advanced miniatured 2 photon calcium imaging in freely behaving mice with over 100 cells collected simultaneously in each session, it provides a powerful tool to investigate the population coding of MEC cells [41].

In this study, we investigate whether MEC grid cells encode past paths and how such coding integrates with existing spatial representations [36, 42]. We address these unresolved questions in our studies: (1) Whether discrete populations of grid cells encode past positions; (2) The relative contributions of visual and self‐motion inputs to this process; (3) Whether the retrospective coding facilitates accurate navigation.

## 2 Results

### 2.1 Retrospective Grid Coding in Mouse Medial Entorhinal Cortex

**FIGURE 1:**
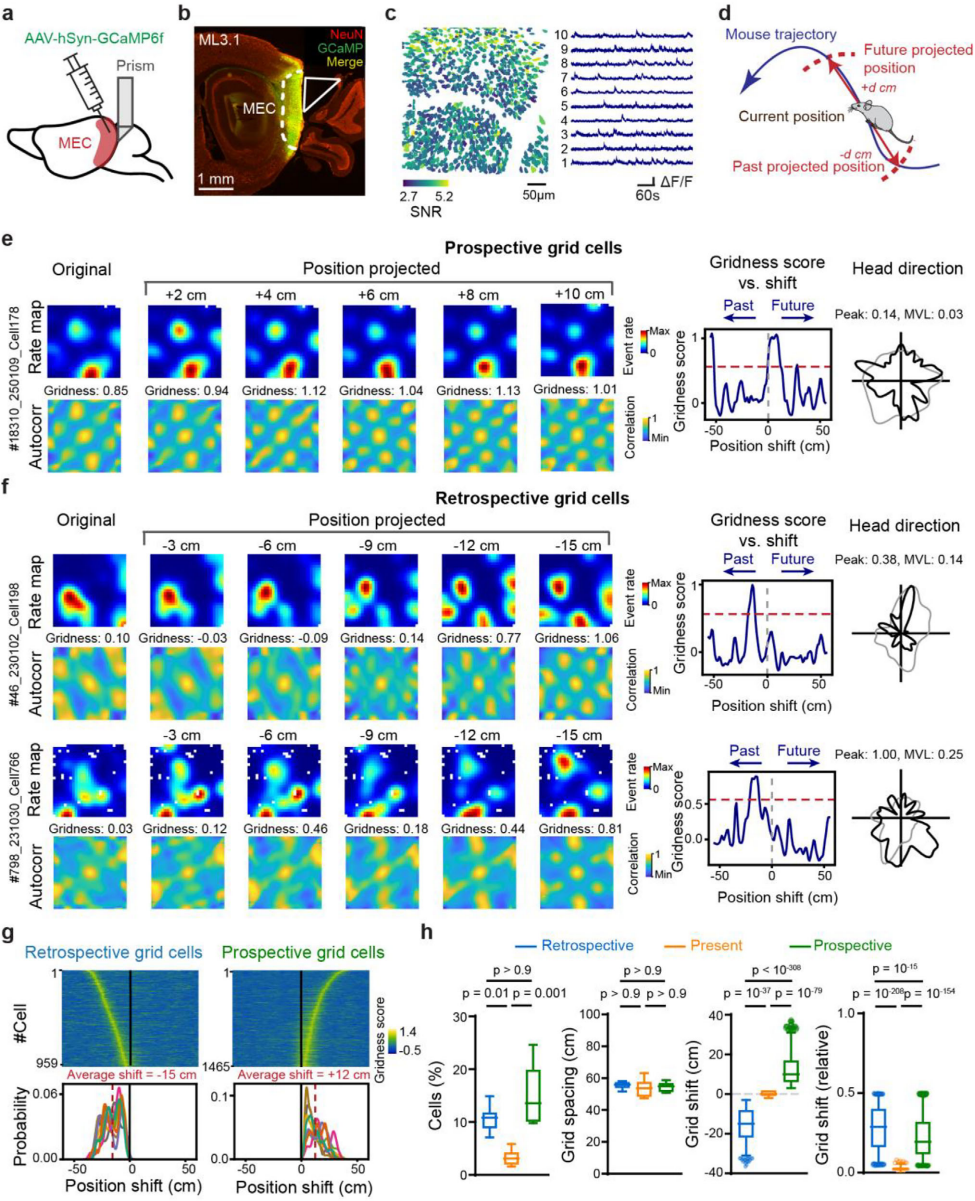
Retrospective grid coding in mouse medial entorhinal cortex. (a) Schematics of virus injection and prism implantation in MEC. (b) Representative example sagittal brain slice showing the position of the prism (white lines). Red, NeuN; green, GCaMP expression; ML: medio‐lateral coordinate. (c) (Left) Representative imaging FOV with a total of 1,036 extracted cells in a single session, colored by SNR. (Right) Example calcium traces (normalized ΔF/F, black traces) for 10 example cells. (d) Schematic illustrates the method used to construct prospective and retrospective maps, in which the positions of the mouse were projected forward or backward, respectively. Mouse schematic from scidraw.io. (e) Firing patterns of a representative prospective grid cell. (Left) Spatial rate maps (top, color‐coded) and autocorrelograms (bottom, color‐coded) at the original position and at different forward‐shifted positions. Gridness, gridness score. (Middle) Gridness scores by the shifted positions, where positive and negative shifts indicate forward and backward projections, respectively. Red dashed line, threshold of gridness score to identify grid cells; gray dashed line, original position. (Right) Head direction tuning curves (black) together with directional behavior (gray) at the original position. MVL, mean vector length; peak, peak event rate in Hz. (f) Same as (e) but for two example retrospective grid cells. (g) (Top) Gridness scores of all retrospective (left) and prospective (right) grid cells by shifted positions (color coded, *n* = 959 and 1465 cells from 8 animals). Black line, original position. (Bottom) Proportion of retrospective and prospective grid cells by shifted positions across animals (color‐coded). Red dashed line, average optimal position shift of all retrospective and prospective grid cells, respectively. (h) From left to right: proportion, grid spacing, position shift and optimal position shift relative to grid spacing of all retrospective, present and prospective grid cells (Friedman test across animals, the fraction of cells: *S* = 12.25, *p* = 0.001, spacing: *S* = 0.75, *p* = 0.7; Kruskal‐Wallis test across cells, shift: *H* = 2246, *p* < 10^−308^, normalized shift: *H* = 974.8, *p* = 10^−211^). For all panels and Figures, boxes: i.q.r., whiskers: ± 1.5×i.q.r., where i.q.r. is the interquartile range.

To investigate the temporal dynamics of spatial coding in the medial entorhinal cortex, we injected an adeno‐associated virus (AAV) encoding GCaMP6f into the MEC and implanted a micro‐prism for chronic imaging (Figure 1a). Large‐scale 2 photon calcium imaging was then performed in freely moving mice using miniatured 2 photon microscopy, enabling population‐level analysis and the identification of distinct grid cell coding properties. Histological examination confirmed precise prism placement (Figure 1b), allowing stable imaging from layer 2 and 3 of MEC. From a representative field of view (FOV), we extracted 1,036 neurons in a single session, with calcium transients (ΔF/F) exhibiting robust signal‐to‐noise ratios (SNR) across cells (Figure 1c).

We implemented a projection method [32] to assess whether grid cells encode future (prospective) or past (retrospective) positions (Figure 1d). To distinguish retrospective grid cells from present grid cells, we established a classification framework based on spatial periodicity. Across all imaged neurons (*n* = 9266 neurons; Figure S1a), grid cells were identified using the 95th percentile of the shuffled gridness score distribution as the significance threshold (dashed red line, Figure S1c; shuffle test, *p* < 0.05). Among all cells exceeding the threshold, only a small proportion (12.4%) showed gridness score peaks within 3 cm of the original position, and these were classified as present grid cells. The remaining cells with peaks at either backward‐ or forward‐shifted positions and surpassing the threshold were designated as retrospective (36.4%) and prospective (51.2%) grid cells (Figure S1d). Retrospective and prospective properties were also observed in cells that surpassed the threshold at the original position (Figure S1e). Comparisons with non‐grid cells and shuffled controls revealed distinct class proportions, arguing against the possibility that the observed distributional structure arose from the analysis artifact (Figure S2a,b). Moreover, sensitivity analyses varying the smoothing parameter and central classification window indicated that the underlying shift structure and most cell classifications remained stable under moderate parameter variations and were not contingent on a specific parameter choice (Figure S2c,d). Further analyses showed that, although some cells exhibited multiple local maxima and across‐day variability, their dominant optimal‐shift preferences were largely preserved (Figure S2e–k).

Prospective grid cells (Figure 1e; *n* = 1465 from 34 sessions) exhibited stable spatial periodicity in rate maps (gridness score > 0.56, *p* < 0.05, shuffle test) at forward‐shifted positions, consistent with previous study [32], but showed only weak head direction tuning (MVL mean ± s.e.m.: 0.20 ± 0.003). In contrast, a distinct population of cells (*n* = 959) displayed maximal gridness score at backward‐shifted positions with hexagonal firing fields (Figure 1f). Such cells were observed in multiple recordings across animals (Figure S3) and were thus termed retrospective grid cells. In parallel with improvements in gridness score, spatial information also increased at the backward‐shifted locations, reinforcing that retrospective grid cells conveyed more information about past locations (Figure S1f). Their optimal shifts (mean ± s.e.m.: −15.66 ± 0.26 cm, Figure 1g, red dashed line) differed significantly from those of prospective cells (mean ± s.e.m.: +12.36 ± 0.20 cm) and present grid cells (mean ± s.e.m.: +0.34 ± 0.07, Figure 1h). Retrospective cells constituted 10.7% of all recorded neurons (Figure 1h, left), with comparable grid spacing across groups (retrospective: 56.46 ± 0.61 cm; present: 54.45 ± 1.61 cm; prospective: 55.51 ± 0.74 cm; Figure 1h). However, retrospective grid cells exhibited significantly larger relative grid shifts (grid shifts divided by grid spacing) than prospective cells (*p* = 10^−15^, Figure 1h, right), with the latter being comparable to values reported previously during free foraging [32]. To exclude the possibility that the shift of retrospective grid cells is caused by slow dynamics in calcium imaging, we examined the behavioral correlates of position shifts in our data. The average optimal shift distance (Figure 1g) aligned with the distance mice traversed in 4.4 ± 0.03 s (Figure S1g, dashed line). This timescale greatly exceeds the imaging timescale (∼0.1 s at 9.5 Hz), suggesting that retrospective coding is unlikely to result from the temporal resolution of calcium imaging. To validate this result using datasets with higher temporal resolution, we analyzed recordings from a subset of animals implanted with tetrodes (Figure S1b) together with a public dataset [43]. Retrospective grid cells were observed in both datasets (Figures S4–S7) and preserved across days in the same environment (Figure S6), albeit with a slight deviation in the average optimal shift (Figure S4c). To further consolidate these findings, we applied a generalized linear model (GLM) to assess how well spike trains could be predicted by positions across different spatial shifts (Figure S7a,b). Both prospective and retrospective grid cells exhibited strong correlation between gridness score and either the explained deviance from GLM or spatial information across position shifts compared to chance (Figure S7c–f), further supporting the robustness of our analysis. Using a public dataset [44], we found that retrospective grid cells could also be identified in the rat medial entorhinal cortex. These cells were phase‐locked to either the troughs or peaks of theta oscillations, but showed no significant difference in shift distance between groups (Figure S8). Together, these findings confirm the presence of retrospective grid coding in the entorhinal cortex.

To dissociate whether retrospective coding reflects spatial or temporal displacement, we compared grid cell activity under position‐shifted and time‐shifted conditions. A representative retrospective grid cell (Figure S9a) exhibited robust hexagonal firing patterns with gridness scores exceeding the threshold (red dashed line) only at backward‐shifted positions but not in the time domain. While the peak gridness score occurred at −15.66 ± 0.26 cm (Figure 1g) with spatial shift, the maximal gridness score emerged at −2.52 ± 0.04 s (*n* = 959 cells, Figure S9b) when applying temporal shifts, suggesting complementary time‐lagged dynamics. This aligns with the animals’ running speed (∼6.0 cm s^−1^), as the average spatial shift (−15.66 cm) and temporal shift (−2.52 s) approximate equivalent displacements. However, despite the presence of retrospective and prospective grid coding during temporal shifts, both gridness score and spatial information were generally reduced at the optimal temporal shift compared with position‐shift analyses (Figure S9c). The stronger spatial than temporal modulation implies that retrospective coding primarily represents path integration over distance, with time acting as a secondary correlate.

### 2.2 Sensory Input Deprivation Disrupts Retrospective Grid Coding

**FIGURE 2:**
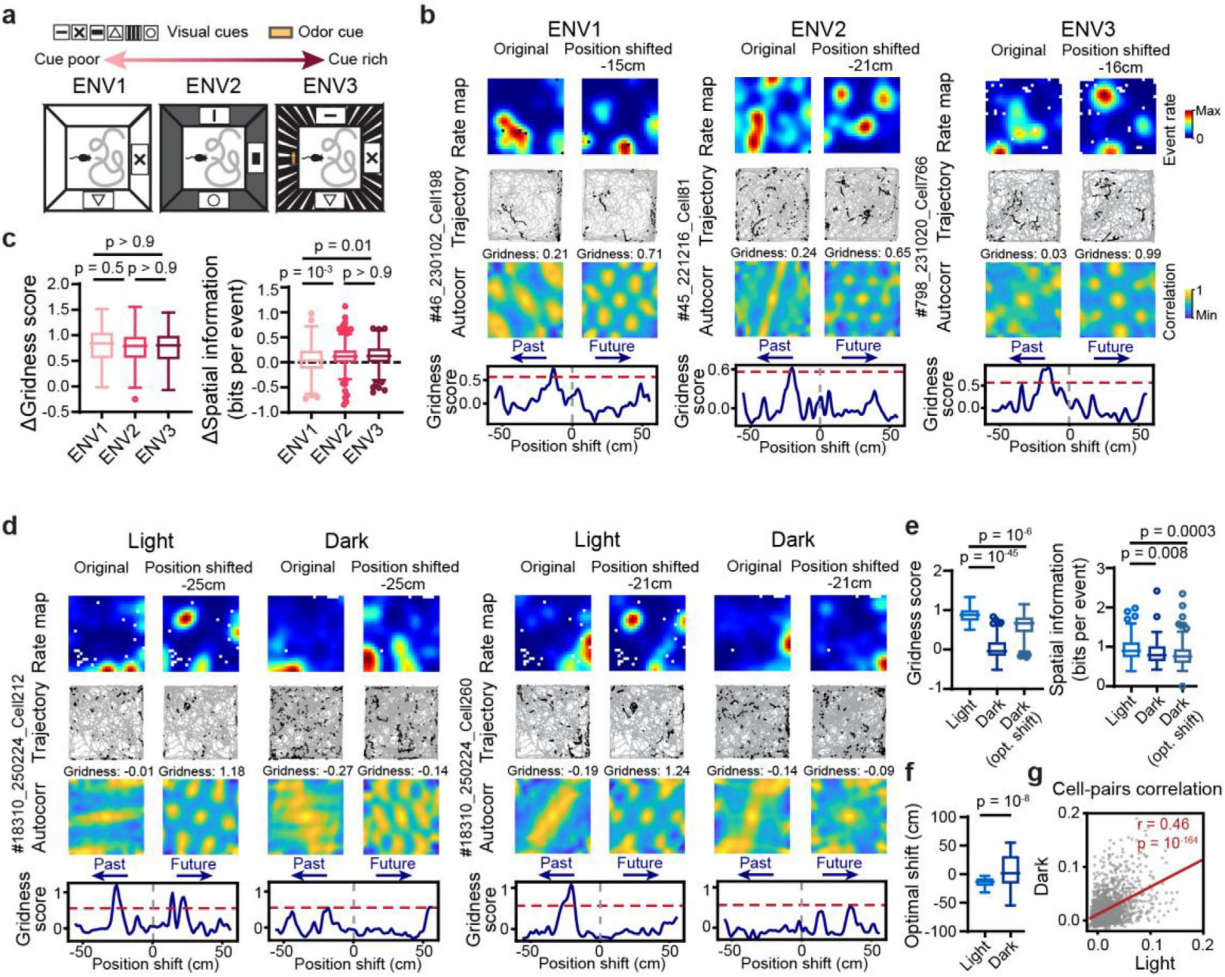
Sensory input deprivation disrupts retrospective grid coding. (a) Schematic of sensory cues across three environments, sorted by cue richness from cue‐poor (left) to cue‐rich (right). (b) Representative firing patterns of three retrospective grid cells in different environments. Spatial rate maps (row 1, color‐coded), event maps (row 2, path in gray and events in black dots), and auto‐correlograms (row 3, color‐coded) at original and backward‐shifted positions. Row 4 shows gridness scores by the shifted positions, where positive and negative shifts indicate forward and backward projections, respectively. Red dashed line, threshold of gridness score to identify grid cells; gray dashed line, original position. (c) Distribution across retrospective grid cells identified under different environments shows improved gridness score (left) and spatial information (right) at the optimal backward‐shifted positions compared to the original positions (*n* = 257, 505 and 197 cells for ENV1, ENV2 and ENV3, respectively; Kruskal‐Wallis test across cells, Δgridness score: *H* = 3.2, *p* = 0.2, Δspatial information: *H* = 121.1, *p* = 10^−25^). (d) Same as (b) but for two representative retrospective grid cells under light and dark conditions. (e) Distribution of gridness scores (left) and spatial information (right) across all retrospective grid cells (*n* = 112 cells, Kruskal‐Wallis test with p‐values indicated). For “Light” and “Dark”, metrics were computed using the optimal shift identified during the light condition; for “Dark (opt. shift)”, the optimal shift was determined separately from the dark condition. (f) Distributions across retrospective grid cells of optimal position shifts under light and dark conditions (two‐tailed Wilcoxon matched‐pairs signed‐rank test p‐values indicated). (g) For each retrospective grid cell pair, the Pearson correlation between their calcium activity traces under light and dark conditions. The red line indicates the linear regression across cell‐pair correlations (Pearson correlation coefficient and p‐value indicated).

Since sensory input is thought to be crucial for maintaining the stability and accuracy of the grid network [36, 37], we asked whether retrospective grid coding also relied on this input. We compared neuronal activity across three environments containing different combinations of discrete sensory cues and optic‐flow information, with comparable spatial coverage across environments (Figure 2a, Figure S10a). ENV2 contained one additional visual cue relative to ENV1, whereas ENV3 additionally contained an olfactory cue and visual gratings relative to ENV2. Retrospective grid cells were detected in all three environments (Figure 2b) and showed comparable improvements in gridness scores (Figure 2c). By contrast, environments containing richer visual cues showed greater increases in spatial information following positional projection (Figure 2c). The additional olfactory and optic‐flow cues in ENV3 produced no further detectable increase relative to ENV2. These results suggest that richer visual cue configurations are associated with greater spatial precision of retrospective representations. However, neither sensory cue richness nor optical flow affected the relative grid shifts of these cells (Figure S10b).

These retrospective grid cells degraded during darkness (Figure 2d). The gridness scores of retrospective grid cells decreased by 95% in darkness (light: 0.88 ± 0.02; dark: 0.04 ± 0.02; dark (opt. shift): 0.62 ± 0.03; *p* = 10^−45^, Kruskal‐Wallis test; Figure 2e, Figure S10c) and only 8% of cells maintained gridness scores above threshold in darkness (vs. 100% in light). Their spatial information dropped by 11% (*p* = 0.0003; Figure 2e, Figure S10c) in the dark, consistent with a reduction in directional tuning, as reflected by a 26% decrease in MVL (*p* = 10^−5^). In darkness, retrospective grid cells identified under light conditions no longer maintained backward optimal shifts; instead, their optimal shifts were distributed around zero (*p* = 0.9; Figure 2f) and did not differ significantly in rank from the shuffled optimal‐shift values (two‐sided Mann‐Whitney test, *p* = 0.14). To further probe population dynamics, we also examined the population‐level stability and the pairwise correlation of calcium activity traces. We found a strong correlation between light and dark conditions (mean *r* = 0.46, *p* = 10^−164^; Figure 2g), which was significantly higher than the shuffled distribution (Figure S10d), indicating that the relationships between retrospective cell pairs are preserved even when the network is decoupled from external sensory inputs. Similar to retrospective grid cells, prospective grid cells also showed degradation, but maintained their coupling under poor visual input (Figure S11). This was not caused by behavior changes in darkness, as animals maintained similar exploration patterns in light vs. dark conditions (Figure S12a), with no significant difference in spatial coverage (light: 87.36% ± 2.70% vs. dark: 92.42% ± 1.65%; *p* = 0.1, Figure S12b) or running speed (light: 10.05 ± 0.64 cm/s vs. dark: 10.61 ± 0.75 cm/s; *p* = 0.4, Figure S12b). Consistent with previous studies [36, 45, 46, 47], the visual modulation was also observed in grid cell population (Figure S12c–e). Similar conclusions were obtained when analyzing across sessions (Figure S13). These results together suggest that sensory information is essential for retrospective and prospective grid coding in the entorhinal cortex.

### 2.3 Assisted Movement Disrupts Retrospective Grid Coding

**FIGURE 3:**
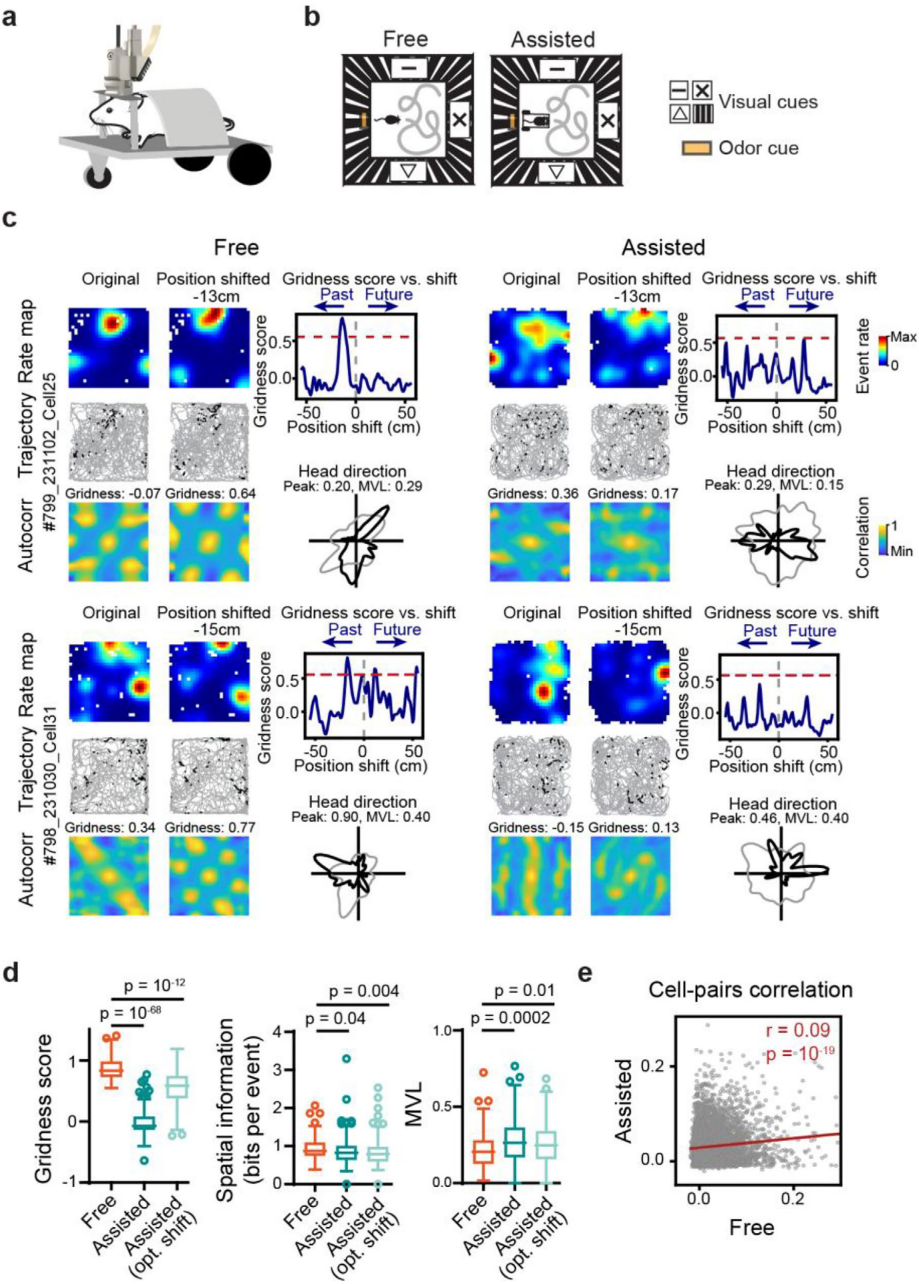
Assisted movement disrupts retrospective grid coding. (a) Schematics of the assisted navigation setup. Mouse schematic from scidraw.io. (b) Mice freely explored an 80‐cm square arena (left), followed by assisted movement via an automatically driven car in the same environment (right). (c) Example firing patterns of two retrospective grid cells during free exploration and assisted movement. (Left) Spatial rate maps (top, color‐coded), event maps (center, path in gray and events in black dots), and auto‐correlograms (bottom, color‐coded) at original and backward‐shifted positions. (Right top) Gridness scores at the shifted positions, where positive and negative shifts indicate forward and backward projections, respectively. Red dashed line, threshold of gridness score to identify grid cells; gray dashed line, original position. (Right bottom) Head direction tuning curves (black) together with directional behavior (gray) at the original position. (d) Distribution of gridness score (left), spatial information (middle), and MVL (right) across all retrospective grid cells (*n* = 151 cells, Kruskal‐Wallis test with p‐values indicated). For “Free” and “Assisted”, metrics were computed using the optimal shift identified during free foraging; for “Assisted (opt. shift)”, the optimal shift was determined separately from the assisted session. (e) For each retrospective grid cell pair, the Pearson correlation between their calcium activity traces under free and assisted conditions. The red line indicates the linear regression across cell‐pair correlations (Pearson correlation coefficient and p‐value indicated).

To examine whether retrospective grid coding differs between active exploration and assisted movement, we compared the activity of the same neurons during free foraging and passive motorized transport within the same 80 × 80 cm arena (Figure 3a,b). While arena coverage and average moving speed were comparable between free‐moving and assisted sessions (Figure S14a,b), we found that the retrospective coding was disrupted during assisted movement (Figure 3c). In the free exploration condition, the retrospective grid cells exhibited robust hexagonal firing patterns at backward‐shifted positions (gridness score: 0.86 ± 0.02) with moderate head direction tuning (MVL: 0.22 ± 0.01). However, in the assisted movement condition, the retrospective grid cells completely lost their grid periodicity (gridness score change: −102%, *p* = 10^−68^), but showed increased directional tuning (MVL change: 131%). The preserved head direction tuning during assisted movement suggests the vestibular inputs remain intact during the assisted session (Figure 3d), while the selective loss of grid coding implicates the breakdown of spatial updating mechanisms in the assisted session. Assisted movement did not reduce the overall magnitude of pairwise correlations among retrospective grid cells (Figure S14c). However, in contrast to the pattern observed after loss of visual input, the correspondence between pairwise correlation coefficients measured during free and assisted movement was not significantly greater than that expected from the shuffled distribution (Figure 3e; Figure S14d). This result suggests a reorganization of the functional correlation structure between conditions. Spatial coding by present [48] and prospective grid cells was also disrupted under assisted navigation (Figures S14e–g, S15, and S16)

### 2.4 Incorporating Retrospective Information Reduces Path Integration Error in an Attractor Network Model

**FIGURE 4:**
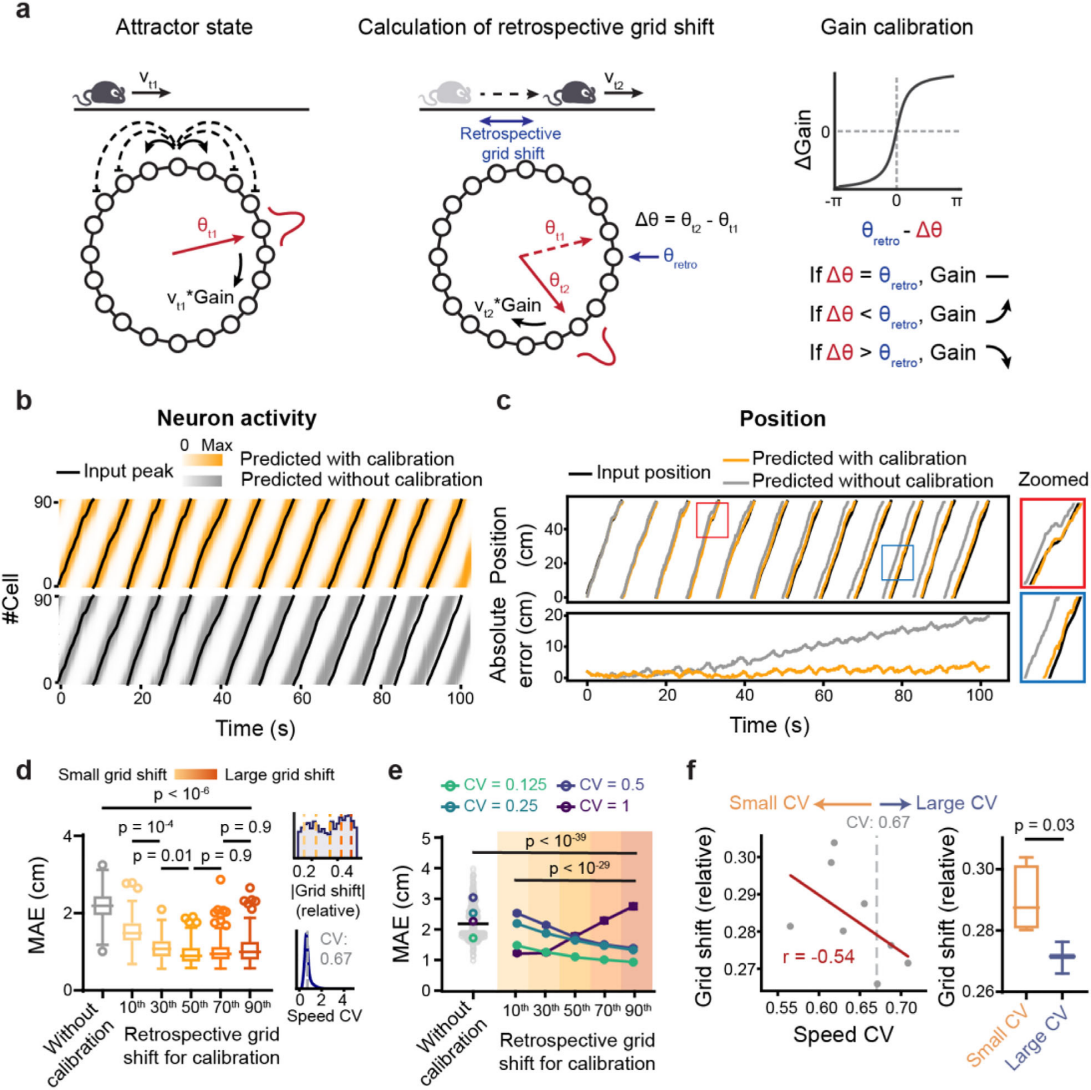
Attractor‐network model with retrospective input calibrates path‐integration. (a) Schematics of path‐integration gain calibration via retrospective input. (Left) As the animal runs along a linear track, the grid system is modelled as a one‐dimensional ring attractor. Grid cells are arranged in a circle based on their firing fields and interconnected by short‐range excitation and long‐range inhibition. The network generates a series of ‘bumps’ as the animal moves, with peak activity at time t represented by phase angle θ_t_, which shifts around the attractor at a rate of v_t_^*^ Gain. (Middle) As the animal moves, changes in attractor phase (Δθ) are compared with the retrospective phase (θ_retro_, blue arrow), which depends on the optimal grid shift. (Right) During gain calibration, the gain is increased when θ_retro_ > Δθ, and decreased when θ_retro_ < Δθ. (b) Peak of simulated neural activity (black) and predicted neural activity (color‐coded), with (top) and without (bottom) retrospective input for path‐integration gain calibration (representative examples). (c) (Left) For examples in b, simulated (top, black) and predicted positions (top, color), with and without gain calibration, along with corresponding absolute error (bottom) across time. (Right) Zoomed view. (d) (Left) Distribution of Mean Absolute Error (MAE) across 100 replications when predicting without calibration or using calibration based on retrospective grid cells with varying grid shifts (Friedman test with p‐values indicated). (Right top) Distribution across retrospective grid cells of absolute optimal position shift relative to grid spacing. Dashed lines: 10th, 30th, 50th, 70th, and 90th percentile of the relative grid shift. (Right bottom) Distribution of the coefficient of variation of speeds in recorded data. The model used the average CV across all frames, indicated by the dashed line. (e) Same as (d) but for MAE calculated by models with different CVs (color‐coded; Friedman test with p‐values indicated). Colored circles, average values across replications for each CV. (f) (Left) For each animal, the average relative grid shift across all retrospective grid cells and their speed CV in recorded data (*n* = 8 animals). Red line, the linear regression (Pearson correlation coefficient indicated). Dashed line, the threshold used to divide animals into small and large CV groups. (Right) Distribution of average relative grid shift across animals in the small and large CV groups (Mann‐Whitney test with p‐values indicated).

Since the retrospective grid network relied on internal proprioceptive signals for maintenance, it may be updated by the path integration‐based mechanism. We hypothesize that the unique properties of retrospective grid cells allow them to calibrate path integration by integrating past experiences, possibly through alternating the speed gain [49]. To computationally validate this hypothesis, we built a one‐dimensional ring attractor network of grid cells following previous models [50]. The model arranged grid cells in a network of neurons with short‐range excitation and long‐range inhibition (Figure 4a, left; Methods). The interconnections between grid cells generate a ‘bump’ of activity, which moves along the continuous attractor as the animal moves. We introduced a retrospective phase (θ_*retro*_), drawn from the distribution of optimal grid shifts in the experimental data, and compared it with the shift in activity bump (Δθ) as the animal traverses this phase. The gain value was then adjusted to minimize the difference, aligning the strength of the velocity input with the actual movement, thereby improving the accuracy of path integration (Figure 4a, middle and right). The change in gain value (δ_*g*_) was calibrated using the following function:

$$\delta_{g}=-0.1\cdot \tanh\left(\frac{\left|\theta_{retro}-\Delta \theta \right|}{20}\right)+1$$

With calibration from retrospective input, the predicted neuron activity better matched the simulated ones (Figure 4b), with lower accumulated absolute error across time compared to the model without calibration (Figure 4c).

As the optimal shift of retrospective grid cells in experimental data forms a continuous distribution (Figure 1g), we further asked whether their contribution to gain calibration varied across this distribution. We generated five models based on optimal shifts derived from different percentiles of the distribution (Figure 4d, right top), with animal's moving speed simulated using the average speed and coefficient of variation (CV) from the experimental data (Figure 4d, right bottom). As expected, the model performed better with calibration, and cells with an average shift contributed the most (Figure 4d, left). Models using different CV values further revealed dynamic shifts in the contributions of cells: cells with smaller shifts contributed more during rapid speed changes, while those with larger shifts contributed during slower changes (Figure 4e). To further test our model, we divided the experimental data into two groups based on the speed CV of each animal. Notably, animals with smaller CV exhibited larger relative retrospective grid shifts, providing further support for our model (Figure 4f). Overall, our model proposes a potential role for retrospective coding during spatial navigation, where cells with varying optimal shifts contribute across different behaviors during foraging, collectively refining the path integration process to enable accurate spatial representation and navigation of the animal in dynamic environments.

## 3 Discussion

Our main finding is the identification of a group of neurons in MEC that exhibit hexagonal firing patterns exhibiting peaks at backward‐shifted positions. These retrospective grid cells require both external visual cues and self‐motion inputs, as their spatial periodicity is disrupted under darkness or assisted movement, similar to present [36, 45, 48, 51] and prospective grid cells [32]. The substantial presence of retrospective grid cells, along with our computational modeling, indicates that MEC stores recently traversed spatial information and utilizes it to correct accumulated errors during path integration, thereby supporting accurate spatial navigation.

Previous studies have demonstrated the presence of prospective and retrospective information in MEC and hippocampal CA1 [18, 26, 27, 28, 29, 30, 31, 32, 34, 52, 53, 54, 55, 56, 57]. Retrospective memory processing has often been investigated in the context of replay and consolidation during rest [8, 58]. Our findings extend this framework by showing that retrospective coding also occurs during ongoing navigation. Specifically, we found that retrospective information was organized into hexagonal firing patterns in mice, which may contribute to accurate navigation. Offline replay and online retrospective grid coding may therefore represent complementary modes of retrospective neural processing. Previous work focused on prospective coding and did not report such retrospective firing patterns [27, 32], which may be attributable to two reasons: (1) species differences, as those studies used rats whereas we used mice; and (2) technological limitations, as prior studies mostly relied on silicon probes (128 channels) or tetrodes, which constrain the number of simultaneously recorded neurons and may miss less abundant cell types. In contrast, our large‐scale two‐photon imaging enables simultaneous recording from hundreds of neurons, increasing the likelihood of detecting such signals. These retrospective representations may be relayed to CA1 or transmitted through the DG/CA3 pathway, although further experiments and analyses are required to clarify the underlying pathways.

The mechanisms underlying the formation of retrospective and prospective grid cells remain an open question. Self‐motion signals, including speed and head direction information [31, 59], contribute to the generation and maintenance of present grid cell firing [48, 60, 61]. Running speed is represented by multiple signals in the MEC, including the activity of speed cells, which have been proposed as a potential source of velocity‐related input to grid‐network dynamics [31, 60]. Temporal information is also represented in the entorhinal system; MEC neurons encode elapsed time during immobility, whereas grid cells jointly encode elapsed time and distance during running in place [62, 63]. In our model, speed and time information were combined to determine the retrospective spatial offset, and incorporating retrospective information reduced accumulated position error across different moving speeds, suggesting a potential computational interaction between velocity and temporal signals. Although retrospective grid coding was disrupted during assisted navigation, this paradigm simultaneously altered multiple sensorimotor and behavioral factors, such as motor efference, proprioceptive feedback, and vestibular and head direction signals. Future studies that independently manipulate these signals will be required to determine their respective contributions and how they are integrated to support retrospective grid coding.

In addition to these internally generated signals, external sensory cues are known to contribute to the formation, stability, and error correction of present grid representations [37, 45, 51, 64]. Our findings suggest that such inputs are also necessary to maintain retrospective grid coding. While sensory deficits, including impairments in vision, hearing, and olfaction, are consistently reported in dementia such as Alzheimer's disease [65, 66, 67, 68] and are associated with degeneration in related neural pathways and pathological changes in brain regions [69], our results suggest a potential functional link between sensory degradation and entorhinal cortex coding. Specifically, disrupted retrospective grid representations under reduced sensory input may contribute to impairments in memory consolidation, a process essential for cognitive function. Besides, the persistence of intercellular coupling in darkness suggests that retrospective grid cells may emerge through mechanisms similar to those underlying present grid cell formation [70, 71], or may originate from present and prospective grid cells [72, 73]. However, further experimental and theoretical investigations will be needed to fully elucidate these mechanisms.

While prospective coding offers a powerful framework for online spatial navigation by generating predictions and updating models through sensory prediction errors [28, 29, 30, 34, 55], it is insufficient for tasks requiring the post‐hoc analysis of past experiences. Retrospective neural coding is essential for several critical navigational functions: (1) Path integration recalibration, where upon encountering a mismatch, the system must retrospectively analyze the recent trajectory to identify the source of error; (2) Cognitive map consolidation, achieved through the offline replay of past sequences in the hippocampus, which strengthens and integrates spatial memories; (3) Route planning and shortcut inference, which relies on the retrospective retrieval and recombination of stored spatial relationships from a cognitive map; and (4) Post‐decision confirmation, such as looking back at a choice point to solidify the spatial context for return trips. Thus, navigation is not solely a forward‐looking process but depends on a dynamic interplay between prospective inference and retrospective evaluation to build a coherent and adaptable spatial model of the world. Here, by incorporating retrospective information, we evaluated the first of these possibilities and showed that the grid network can be driven to the correct phase, with cells exhibiting different optimal shifts contributing across various behaviors. Together, our results offer new insights into how animals may leverage past experiences to refine path integration, providing a dynamic mechanism for flexible spatial navigation.

## 4 Method Section

### 4.1 Animals

A total of 11 adult male C57BL/6N wild‐type mice were used in this study. Animals were individually housed under controlled environmental conditions (20°C, 50% humidity) with a 12 h light/dark cycle. Behavioral experiments were conducted during the dark phase. Food intake was restricted to 2–4 g per day to maintain body weight at approximately 90% of the animals’ free‐feeding level. Mice were chronically implanted in the right hemisphere with either a Microdrive (Axona) with eight tetrodes (*n* = 3) or a prism (*n* = 8). All animals and all recorded data were included for analysis. All animal experiments were approved by the Institutional Animal Care & Use Committee of Peking University.

### 4.2 Virus Injection and Prism Implantation in MEC

Animals were anesthetized with isoflurane (0.5%–3% in air; flow rate: 0.8–1.0 L/min) in a chamber, with the concentration adjusted based on physiological conditions. Once fully anesthetized, animals were head‐fixed on a stereotaxic frame (KOPF) and maintained under 1%–2% isoflurane. Erythromycin ointment was applied to protect the eyes from drying. The hair was shaved, and the skin over the skull was sterilized with 70% ethanol and betadine. A midline incision was then made to expose the skull. A small craniotomy was made above the right MEC, and the dura was carefully removed using a blunt‐tip needle. For virus injection, a total volume of 0.8 µL AAV‐hSyn‐GCaMP6f (titer > 2 × 10^13^ vg/mL; BrainVTA, China) was injected at two sites (ML: +3.1 mm, AP: +0.3 from sinus, DV: −1.8 to −1.4 mm; ML: +3.7 mm, AP: +0.3 from sinus, DV: −1.5 to −1.3 mm) in the MEC using a pulled glass micropipette, delivered at a rate of 30 nL/min using a Nanoliter 2020 injector (World Precision Instruments). Following injection, the pipette was left in place for 10 min to ensure diffusion and minimize backflow.

Next, a 3.5 × 2.5 mm^2^ prism (Grintech) was inserted into the space between the cerebellum and cerebrum, with its flat surface aligned to the dorsoventral surface of the MEC. To stabilize the implant and protect the exposed tissue, the craniotomy was sealed with Kwik‐Sil (World Precision Instruments), followed by the application of tissue adhesive (3 M) to bond the skull to the surrounding scalp. Dental cement (Super Bond) was subsequently applied to secure the implant and cover the remaining exposed skull. A stainless steel headbar (Transcend Vivoscope) was then mounted on the cement surface and aligned parallel to the top face of the prism.

Following surgery, animals recovered on a heated pad until fully awake and then returned to their home cages. They were housed individually and received daily injections of cephalosporin for 1 week to prevent infection.

### 4.3 Miniaturized 2‐Photon Microscope Calcium Imaging

Three weeks after surgery, mice were head‐fixed using an adapter, and an optimal field of view (FOV) was selected using a wide‐field single‐photon microscope to identify regions with maximal neuronal visibility. A baseplate was then secured to the headbar to ensure stable mounting of the imaging system in subsequent sessions.

Calcium imaging was performed using a miniatured two‐photon microscope (LFMTPM V2.0.1), with system control and data acquisition managed via GINKGO‐MTPM software (Transcend Vivoscope Biotech Co., Ltd., China). GCaMP6 fluorescence was excited at 920 nm using a laser (TVS‐FL‐01, Transcend Vivoscope Biotech Co., Ltd China), with a typical laser power of approximately 70 mW at the sample. Images were collected at 9.5 Hz with a spatial resolution of 600 × 512 pixels, corresponding to a field of view of 534 × 455 µm. Considering the location of virus injection and the properties of 2‐photon imaging, the recorded field of view is most likely located in layers 2 and 3 of MEC.

Behavioral tracking and imaging were acquired simultaneously using the same software platform. Mouse trajectories were tracked at 25 Hz using an overhead camera, and the behavioral data were temporally aligned to the imaging timestamps by linear interpolation and subsequently downsampled to match the calcium imaging frame rate (9.5 Hz).

### 4.4 Surgery and Tetrode Implantation in MEC

Electrodes were manually assembled, each comprising four tetrodes (16 recording channels in total). Each tetrode was fabricated by twisting four platinum wires together and heat‐bonding the insulation at approximately 250°C. The complete electrode assembly included four tetrodes, two grounding wires, and a connector module with gold pins. Four screws allowed for independent depth control for each tetrode. The wire tips were then trimmed into spherical shapes. Electrode impedances were reduced to 100–240 kΩ by platinum electroplating using the NanoZ system.

Prior to implantation, mice were anesthetized and placed in a stereotaxic frame. The scalp was removed to expose the skull, and the skull surface was adjusted to ensure a bregma–lambda height difference of less than 0.03 mm. A 0.6 mm circular craniotomy (ML: +3.0 to 3.6 mm) was drilled above the transverse sinus. The electrode was inserted posteriorly at a 5° angle in the sagittal plane at a predefined site (AP: +0.3 mm from sinus; DV: −0.8 to 1.0 mm from dura). Silicone gel and a sponge were wrapped around the electrode to allow controlled vertical movement. Four to five skull screws were inserted for stabilization, with two screws connected to the ground wire. Dental cement was used to secure the implant, and adhesive tape was applied to protect the ground connection.

Postoperatively, mice received intraperitoneal injection of cefazolin (125 mg/mL, 0.1 mL/day) for five consecutive days and were allowed to recover for 3 weeks before recordings began.

### 4.5 Tetrode Recording

Approximately one week following electrode implantation, screws were turned to gradually lower the tetrodes. Data acquisition started around three weeks post‐surgery. During recording sessions, mice were connected to the Axona data acquisition system via an AC‐coupled unity‐gain amplifier. Tetrodes were lowered incrementally in steps of ∼50 µm until well‐isolated single‐unit activity was observed. Recording began once neuronal signals exceeded four times the background noise (typically 20–30 µV) and stable individual units were maintained for over 1 h. Spike signals were amplified between 8000 and 25 000 times, band‐pass filtered from 0.8 to 6.7 kHz, and digitized at a 48 kHz sampling rate (50 samples per waveform, 8 bits/sample), with timestamps recorded at a 96 kHz clock resolution. Local field potentials were amplified 3000–10 000 times, low‐pass filtered at 500 Hz, and digitized at 4.8 kHz.

Animal position and movement were monitored using an overhead video tracking system that recorded the location of head‐mounted LEDs at 50 Hz. All signals were synchronized for subsequent offline spike sorting and behavioral analysis.

### 4.6 Histology

After finishing the experiments, mice were anesthetized via intraperitoneal injection of 0.6 mL 2.5% tribromoethanol and subsequently secured onto a perfusion apparatus. A midline incision was made from the abdomen to the thoracic region using dissecting scissors. A perfusion needle was inserted into the left ventricle following an incision in the right atrium. Animals were perfused with 40 mL of physiological saline, followed by 20 mL of 4% paraformaldehyde (PFA). After perfusion, brains were carefully extracted, post‐fixed in 4% PFA at 4°C for at least 24 h, and then dehydrated in 30% sucrose until fully saturated, as indicated by tissue sinking. Brain tissues were then embedded in OCT Compound (4583, Sakura, Tokyo, Japan) on a metal platform and frozen using a cryostat. Coronal (for hippocampus) or sagittal (for MEC) sections were cut at a thickness of 30 µm.

Sections were rinsed twice with PBS (phosphate‐buttered saline, 300 µL each slide, 5 min each time), followed by incubation in blocking solution containing 10% goat serum and 0.3% Triton X‐100 in PBS for 1 h at room temperature. Primary antibodies–chicken anti‐GFP (Invitrogen) and rabbit anti‐NeuN (Abcam)–were diluted 1:1000 in blocking solution and applied overnight at 4°C. After three PBS washes, sections were incubated for 2–4 h at room temperature with species‐specific secondary antibodies (Alexa Fluor 488, Invitrogen; Alexa Fluor 568, Abcam) and DAPI (Sigma–Aldrich), each diluted 1:1000 in blocking solution. After two final PBS rinses, sections were mounted on glass slides using Fluoromount‐G (SouthernBiotech) and coverslipped.

Fluorescence images were acquired using either a Zeiss Axio Scan Z1 or an Olympus Virtual Slide System to verify the location of implantation and assess virus expression.

For animals implanted with tetrodes, brain sections were mounted onto slides and cresyl violet Nissl‐stained to visualize electrode tracks.

### 4.7 Free Exploration Task

Following complete postoperative recovery, mice were habituated for at least three consecutive days to the open‐field arena used for recording. For calcium‐imaging experiments, animals explored square plastic arenas measuring 80 × 80 cm with 40‐cm‐high walls. ENV1, ENV2, and ENV3 used different boxes located in the same recording room. ENV1 contained two visual cue cards, whereas ENV2 contained three. ENV3 contained three visual cue cards, additional plastic gratings covering the walls, and an olfactory cue consisting of 5% (v/v) ethyl acetate in mineral oil presented on a cotton pad. The cue cards measured 30 × 21 cm, displayed distinct geometric symbols, and were centered at mid‐wall height. For experiments conducted in the dark, the lighting was turned off (peak: 0.036 lux) before placing the mouse in the arena. For tetrode recordings, the arena size was 100 × 100 cm for both our own recordings and the public dataset. For the rat dataset, the arena size was 150 × 150 cm.

During recording sessions, animals freely explored the environment while foraging for small butter cookies (PAUL KEI) that were scattered randomly into the arena at 10–15 s intervals. Calcium imaging or extracellular electrophysiological recordings were conducted during exploration. To enable cross‐condition comparisons of neuronal activity, recordings were performed continuously while animals were exposed to different experimental conditions (light/dark and free/assisted), allowing the same neurons to be tracked across conditions. To minimize residual sensory cues, the arena was thoroughly cleaned between sessions using a dust vacuum followed by 75% ethanol.

The free exploration dataset comprised 34 recording sessions, with recordings obtained from three animals in each environment. ENV1 and ENV2 sessions lasted 60 min, whereas ENV3 sessions lasted 30 min. Different environments were tested on separate days, following a random order across animals. For comparisons across environments, only the first 30 min of each ENV1 and ENV2 session were analyzed to match the duration of ENV3 sessions.

### 4.8 Assisted Navigation Task

Mice were head‐fixed onto a custom‐built, autonomously driven car that navigated within the same open‐field arena. The car was powered by six rechargeable batteries (Delipow, 2700 mA) and driven by two independently controlled motors (WHEELTE), each mounted on one side of the chassis. Motor output was regulated by an Arduino board, enabling differential drive, whereby both motors rotated in the same direction during forward movement and in opposite directions during turning. To ensure stable low‐speed movement, motors with a high reduction ratio were employed.

The car's movement was programmed to mimic the stochastic exploratory patterns of freely moving mice. At each decision point – occurring at random intervals sampled uniformly between 100 and 1500 ms – the system selected a new action with predefined probabilities: 92.5% forward, 7% left or right turn, and 0.5% stop. The selected action was then executed until the next decision point. During forward movement, the ultrasound sensors positioned at the front of the car measured the distance to nearby walls every 100 ms. If a wall was detected within 5 cm, an automatic avoidance response was triggered, prompting the car to briefly reverse and turn away from the wall. Locomotion speed was updated at each decision point, with the new speed drawn from a uniform distribution between 2 and 30 cm s^−1^. Movement speed and head direction were randomized at each time point, while the average speed matched that observed during free foraging. The assisted navigation dataset comprised 5 recording sessions, each lasting approximately 30 min.

### 4.9 Pre‐Processing of 2‐Photon Calcium Signal

Following data acquisition, calcium imaging data were preprocessed offline using *Suite2P74* (https://github.com/MouseLand/suite2p), which performed motion correction, spatial footprint identification, and extraction of fluorescence traces. The resulting data were analyzed using custom MATLAB scripts (R2020a, The MathWorks, Natick, MA) to calculate the relative fluorescence change (ΔF/F) for each ROI^75^. For each ROI, the neuropil‐corrected fluorescence was calculated as:

$$\mathrm{Fraw}\;\left(t\right)=F\left(t\right)-0.7\times Fneu\left(t\right)$$

To correct for slow baseline fluctuations, including photobleaching, a running baseline F0(t) was estimated using the eighth percentile of Fraw(t) within a 30‐s moving window. A constant offset was then added so that Fraw(t) – F0(t) was centered around zero during low‐variance baseline periods. The normalized fluorescence signal was calculated as:

$$\frac{\Delta F}{F}=\left(\mathrm{Fraw}\left(t\right)-F0\left(t\right)\right)/F0\left(t\right)$$

*Δ*F/F traces were subsequently deconvolved using *OASIS76* (https://github.com/zhoupc/OASIS_matlab) to estimate underlying neural activity. Significant calcium transients were defined as periods during which ΔF/F exceeded two standard deviations above the baseline for at least 0.75 s^77^. Within these segments, a transient was classified as a true calcium event if its peak amplitude further exceeded one standard deviation above the baseline level. CellReg 78 (https://github.com/zivlab/CellReg) was used to identify putatively identical cells across days within each reattachment period.

### 4.10 Pre‐Processing of Electrophysiology Data

Spike sorting was conducted using *TINT* (Axona), an offline graphical clustering tool. Manual clustering was performed in two‐dimensional projections of a multi‐parameter space, primarily using waveform amplitude and temporal features.

### 4.11 Tracking of Behavioral Data

Behavioral recordings were analyzed using *DeepLabCut* 79 (https://github.com/DeepLabCut/DeepLabCut). In each frame, four body parts were tracked: nose, neck, left ear, and right ear. The network was trained on 50 manually annotated frames using a resnet_50 backbone for 100 000 iterations. Only position estimates located within the arena boundaries were retained and subsequently smoothed with a 15‐point moving average filter to reduce tracking noise. Head direction was calculated as the orientation vector from the animal's neck to nose.

### 4.12 Spatial and Directional Rate Maps

For all analyses described below, cells with fewer than 50 calcium events or spikes were excluded to avoid potential bias and noise arising from low firing rates. Rate maps were then generated for each cell by normalizing activity to occupancy time. Neuron activity was binned based on behavioral data, using 3 × 3 cm^2^ bins for spatial maps and 3° bins for directional maps, then divided by the occupancy duration within each bin. Rate maps were subsequently smoothed using a 2D Gaussian kernel (σ = 6 cm) for spatial data or a 30° moving average filter for directional data. Spatial firing fields were defined as contiguous regions exceeding the 60th percentile of the peak firing rate, provided the peak rate surpassed 0.5 Hz. To minimize behavioral influences, all data points with running speeds below 2 cm s^−1^ were excluded for analysis.

### 4.13 Analysis of Grid Cells

Spatial autocorrelation analysis was performed for each neuron following established methods [36, 59] to identify grid cells. The spatial autocorrelogram was computed as the correlation between the firing rate map λ(*x*, *y*) and its spatially shifted version λ(*x* − τ_*x*_,*y* − τ_*y*_), defined as:

$$\begin{array}{c} r\left(\tau_{x}-\tau_{y}\right)=\frac{\begin{array}{c} n\Sigma \lambda \left(x-y\right)\lambda \left(x-\tau_{x},y-\tau_{y}\right)\\ -\Sigma \lambda \left(x,y\right)\Sigma \lambda \left(x-\tau_{x},y-\tau_{y}\right) \end{array}}{\begin{array}{c} \sqrt{n\Sigma \lambda {\left(x,y\right)}^{2}}-{\left(\Sigma \lambda \left(x,y\right)\right)}^{2}\\ \times \;\sqrt{n\Sigma \lambda {\left(x-\tau_{x},y-\tau_{y}\right)}^{2}-{\left(\Sigma \lambda \left(x-\tau_{x},y-\tau_{y}\right)\right)}^{2}} \end{array}}, \end{array}$$

where λ(*x*, *y*) denotes the mean firing rate at position (*x*, *y*), and the summations are taken over all η pixels where firing rates exist for both maps. Autocorrelation was only calculated when η ≥ 20.

To quantify hexagonal symmetry in grid cell activity, we adopted the same method as in previous studies (80, [32]). For each cell, the six autocorrelogram peaks closest to the center were identified within 1.5 times the distance to the nearest peak. If fewer than six peaks were detected, all available peaks within the range were included. To compute the gridness score, annular regions were extracted from the autocorrelogram using an inner radius of 30 cm and outer radii ranging from 33 to 120 cm, in 3 cm steps. For each annulus, Pearson's correlations were calculated between the original segment and its rotated versions at 30°, 60°, 90°, 120°, and 150°. The gridness score was defined as the minimum correlation at 60° and 120° minus the maximum correlation at 30°, 90°, and 150°, and the highest score across all annuli was taken as the final gridness score. Grid spacing was measured as the mean distance from the center to the six surrounding peaks in the autocorrelogram. For analyses of retrospective and prospective grid cells, autocorrelograms were computed from firing rate maps aligned to the optimal shift.

Statistical significance was evaluated via a permutation test with 1000 random shuffles per cell. For each permutation, the neuron's activity was circularly time‐shifted along the animal's trajectory by a random offset drawn from a uniform distribution covering the entire session, with a 30‐s exclusion window on either end to eliminate temporal correlations between neural activity and behavior. A neuron was classified as a present grid cell if its gridness score exceeded the 95th percentile of the shuffled distribution.

### 4.14 Analysis of Spatial Cells

Spatial information was computed for each neuron to quantify how well its activity encoded the animal's location (82). It was defined as:

$$I=\Sigma_{i}p_{i}\lambda_{i}\log_{2}\frac{\lambda_{i}}{\lambda },$$

where λ_*i*_ is the mean firing rate of the neuron in the *i^th^* spatial bin, λ is the average firing rate, and *p^i^* is the probability of occupancy in the *i^th^* bin. Spatial stability was assessed by calculating the Pearson correlation between spatial rate maps from the first and second halves of each recording session.

Shuffling was performed similarly. A neuron was classified as spatially tuned if its spatial information exceeded the 95th percentile of the shuffled distribution.

### 4.15 Analysis of Head Direction Cells

To assess head direction selectivity, the MVL was calculated for each neuron. MVL represents the magnitude of the average vector across all directional tuning bins and was computed using the Circular Statistics Toolbox in MATLAB^81^.

Head direction information was used as a complementary measure of directional tuning fidelity. It was calculated as spatial information 82 but applied to angular tuning curves based on directional occupancy and neuron activity. Unlike MVL, head direction information is not biased by the bimodal or multi‐peaked tuning, making it a robust alternative for quantifying directional selectivity.

### 4.16 Identification of Retrospective and Prospective Cells

To classify grid cells as retrospective, present, or prospective, we first computed the gridness score across a series of time‐ or position‐shifted spatial firing maps for each cell. The optimal shift for each cell was defined as the time or position shift at which the maximum gridness score occurred. Cells were included for further analysis only if they met the following criteria: (1) the gridness score at the optimal shift exceeded the 95th percentile threshold; (2) the optimal shift was less than half of the grid spacing (divided by average speed in temporal shift). Based on the optimal shift values, cells were then classified as retrospective, present, or prospective grid cells. Specifically, cells with optimal shifts within 3 cm of the original position were classified as present grid cells, while those with backward‐ or forward‐shifted optimal positions were designated as retrospective and prospective grid cells, respectively. For multiple‐peak detection, peaks were identified using a minimum gridness score of 0.56, a minimum prominence of 0.3, and a minimum interpeak separation of 10 cm. Detected peaks were ranked by gridness score, with the global maximum defined as the primary peak.

### 4.17 Position and Time Projection Method

To compute position‐ or time‐shifted firing maps, neuron activity was reassigned along the animal's trajectory based on a fixed spatial or temporal offset. For each position shift *d*, neuronal activity at each time point was paired with the animal's position at a trajectory distance of |*d*| from its current position. Negative values (*d* < 0) corresponded to previously traversed positions and were defined as retrospective shifts, whereas positive values (*d* > 0) corresponded to positions reached later along the trajectory and were defined as prospective shifts. Firing‐rate maps were then constructed using these reassigned positions. A similar procedure was applied for time shifts, where the projected position was determined based on a temporal offset *t* from the current timepoint.

### 4.18 Generalized Linear Model

We implemented a Poisson generalized linear model (GLM) on electrophysiology data to predict spike trains from the animal's position. Position was first projected onto Gaussian basis functions, yielding 121 spatial features (spatial bin size: 10 cm) at each time point. Each individual session was then split into five folds; four folds were used for training and the remaining fold for testing.

Model performance was evaluated using the explained deviance of the test data in each cross‐validation fold, defined as:

$$\begin{array}{ccc} Explained\;deviance & = & \frac{1}{N}\Sigma_{t=1}^{N}\left[\bar{\mu_{l}}-y_{i}\log\left(\bar{\mu_{l}}\right)\right]\\ & & -\frac{1}{N}\Sigma_{t=1}^{N}\left[{\hat{u}}_{l}-y_{i}\log\left({\hat{u}}_{l}\right)\right], \end{array}$$

where *y_i_* is the observed firing rate at timepoint *i*, ${\hat{u}}_{l}$ is the GLM‐predicted rate, and $\bar{\mu_{l}}$ is the mean observed rate.

### 4.19 Implementation of Attractor Network Model

Grid cells are modeled as a one‐dimensional (1D) periodic network of neurons, velocity‐conjunctive cells, where all neurons are arranged along a circular‐shaped neuron sheet. Following previous studies [50], we assume that all neurons in this 1D network are evenly distributed along the ring, so that each neuron corresponds to a specific location phase. We also introduced 2 additional leftward (*V^L^*) and rightward (*V^R^*) velocity‐conjunctive cells, which are selectively activated during leftward or rightward movements, respectively.

The connectivity between the population within the 1D neuron sheet and the velocity‐conjunctive cells to 1D neuron sheet is determined by learning. In the learning process, the external directional cues (such as landmark information) can activate a localized ‘bump’ of neural activity on this ring, representing the animal's current orientation in space. The animal is assumed to move in consistent speed in both directions.

During the training phase, the activity of 1D attractor units is updated as:

$$\tau \;\frac{dh_{i}\left(t\right)}{dt}=-h_{i}\left(t\right)+\phi \sum_{j}J_{ij}V_{j}+\phi_{r}g\sum_{C\varepsilon W,E}\sum_{k}J_{jk}^{C}V_{k}^{C}V^{C}+I_{i}\left(t\right)$$

where $\phi \propto N^{2}$, $\;\phi_{r}\propto N\cdot N^{C}$. The component *V^C^* scales with the animal's running speed. When the movement direction is leftward, the rightward input *V^R^* is set to zero, and vice versa. The gain value for the speed input is denoted as *g*.

*I_i_* (*t*) is the external cue input to unit *i* with Gaussian‐like tuning:

$$I_{i}\;\left(t\right)=e^{-\beta \left(\Delta \psi^{2}/\sigma_{HD}^{2}\right)}$$

where $\Delta \psi =\;\min(|\psi \left(t\right)-\psi_{i}\left|,L-\right|\psi \left(t\right)-\psi_{i}|)$, ψ(t) is the correct head direction at time t, ψ_*i*_ is the assumed preferred direction of cell i. L is the neural sheet periodicity of the 1D neuron sheet.

The connectivity between unit i and unit j in the 1D neuron sheet is updated according to the Hebb‐like rule:

$$\delta \;J_{ij}=kr_{i}r_{j}$$

The connectivity between the conjunctive unit in the 1D neuron sheet and unit j is updated as:

$$\delta \;J_{ij}^{C}=k_{C}\;r_{i}r_{j}r_{C}$$

The connectivity is normalized by its Frobenius norm for both J and *J^C^*. The firing rate of the 1D attractor unit is a sigmoid function of activation h:

$$V_{i}\;\left(t\right)=\frac{1}{1+e^{-2\beta \left(h_{i}\left(t\right)-\alpha \right)}}$$

The activity of velocity‐conjunctive cells is:

$$V_{j}^{C}=e^{-\beta \left(\Delta \psi^{2}/\sigma_{HD}^{2}\right)}$$

During the testing phase, the learned connection weights are fixed. Neural activity now depends only on the incoming velocity signals, allowing the network to perform path integration. The input to the 1D neuron sheet unit i is:

$$\tau \;\frac{dh_{i}\left(t\right)}{dt}=-h_{i}\left(t\right)+\phi J_{ij}V_{j}+\phi_{r}g\sum_{C\varepsilon W,E}J_{ij}^{C}V_{j}V^{C}$$

During testing, we examine how the model performs under variable‐speed conditions and with additional retrospective inputs as calibration. For a calibration with distance *d*_0_, at each step we search back from the current time point t to a past time *t*_0_ when the animal had traveled a distance *d*_0_. If the integrated distance d between *t*_0_ and t satisfied d < *d*_0_, we increase the velocity gain g; if d > *d*_0_, we decrease g. The change in δ_*g*_ follows a sigmoid function of δ_*d*_:

$$\delta_{g}=-0.1\times \tanh\left(\frac{\left|d_{0}-d\right|}{20}\right)+1$$

To determine the initial gain value in each condition, we searched over a range of gain values around the initial estimate and performed 100 path integration trials, each consisting of 300 steps (without any correction). The gain value that produced the smallest mean integration error was selected as the initial gain for that particular velocity configuration.

To explore how variability in self‐motion signals affects performance, we draw the velocity inputs from a log‐normal distribution with the same mean value and different coefficient of variations (CVs). The CVs of the behavioral data were calculated within consecutive 1 s windows as the standard deviation of speed divided by the mean speed. For each CV condition, we tested five different retrospective shifts, corresponding to the 10th, 30th, 50th, 70th, and 90th percentiles of actual behavioral data. Each trial consists of around 500 steps to match the real time period of 30 s. The time duration of each step is set to make the mean time for agent travel for one period match the real time for the animal travel for one period. The length of the period of the 1D neuron sheet is set to match the actual mean spacing of the real grid cells. Model performance was quantified using the MAE between the simulated and the predicted positions across all time points.

## Author Contributions

**Ruojin Liu**: writing – review and editing, writing – original draft, formal analysis, data curation, methodology. **Xin Yuan**: formal analysis, writing – review and editing, methodology, writing – original draft. **Zilong He**: formal analysis, methodology. **Hong Jin**: data curation. **Jiayi Tian**: data curation. **Cong Chen**: data curation. **Yuanjing Liu**: data curation. **Xiang Zhang**: methodology. **Shidan Wen**: data curation, methodology. **Emilio Kropff**: supervision. **Chenglin Miao**: writing – original draft, writing – review and editing, project administration, supervision, funding acquisition, conceptualization.

## Conflicts of Interest

The authors declare no conflicts of interest.

## Supporting information




**Supporting File**: advs77884‐sup‐0001‐SuppMat.docx.

## Data Availability

The data that support the findings of this study are available from the corresponding author upon reasonable request.
