# Supplementary material for: Retrospective Grid Cells in the Medial Entorhinal Cortex Encode Past Paths During Spatial Navigation
Source: Adv Sci (Weinh). 2026 Sep 27:e77884. Online ahead of print. doi: 10.1002/advs.77884 (PMC13616325; doi:10.1002/advs.77884)
Supplement: Supplementary file 1 [file ADVS-9999-e77884-s001.docx]

**Supplementary Figure 1. Classification and coding properties of retrospective grid cells.**

**(a)** Distribution of cell count per animal (left), session counts per animal (middle), and cell counts per session (right) for the imaging dataset. **(b)** Same as in (a) but for the electrophysiology dataset. **(c)** Distribution of shuffled gridness scores (gray) across the pool of all cells. Dashed red line, 95^th^ percentile. **(d)** Cells were classified as retrospective, present or prospective grid cells based on the position shift at which the maximum gridness score was observed. **(e)** (Left) Proportion of cells across sessions with gridness scores exceeded the threshold at the original position. (Right) Distribution of optimal shifts for these cells, with inset showing their classification by optimal shift. **(f)** Distribution of spatial information, mean vector length (MVL) and head direction information across all retrospective grid cells, shown at both the original and optimal backward-shifted positions (two-tailed Wilcoxon matched-pairs signed rank test p-values indicated). **(g)** Cumulative distribution of traveled distance over time across all the animals (mean ± s.e.m.). Animals typically took at least 4.4 seconds to traverse the average optimal position shift (dashed line).

**Supplementary Figure 2. Robustness of grid cell classification.**

**(a)** Proportions across sessions of retrospective, present and prospective classifications among recorded grid cells, non-grid cells and shuffled controls (left), and distributions of their relative grid shift (right) (Kruskal-Wallis tests with p-values indicated). The same classification boundaries of -3 and +3 cm were applied to all three groups. **(b)** Same as (a) after excluding cells whose absolute optimal shifts exceeded half of the corresponding grid spacing. Left, proportions across sessions of retrospective, present and prospective classifications; right, maximum gridness scores at the optimal shifts of the remaining cells (Kruskal-Wallis tests with p-values indicated). **(c)** Sensitivity of the classification to the smoothing parameter. Left, proportions of retrospective, present and prospective grid cells across smoothing values (mean ± s.e.m., across sessions). Middle, Pearson correlation between each cell’s gridness-versus-shift curve obtained at each smoothing value and that obtained using the original parameter (σ = 1). Right, mean absolute change in optimal shift relative to that obtained using σ = 1. The middle and right panels show mean ± s.e.m. across cells classified using the original parameters. The red dashed line indicates the original smoothing parameter (σ = 1). **(d)** Proportions of retrospective, present and prospective grid cells obtained using different central classification window boundaries (mean ± s.e.m., across sessions). The red dashed line indicates the original boundary (± 3 cm). **(e)** Proportions of retrospective, present and prospective grid cells exhibiting multiple detected peaks. **(f)** Distribution of the relative grid shift of the primary and second-ranked peaks in retrospective, present and prospective grid cells (two-sided Mann-Whitney test with p-values indicated). **(g)** Same as (e) but after excluding peaks whose absolute positional shift exceeded half of the corresponding grid spacing. **(h)** Gridness scores of the primary and second-ranked peaks in retrospective, present and prospective grid cells, before and after application of the grid spacing criterion (Kruskal-Wallis tests with p-values indicated). **(i)** Distributions of next-day optimal shifts for cells classified as retrospective, present and prospective grid cells on the preceding day. Arrows indicate the peaks of the distribution. **(j)** Distributions of the absolute changes in optimal shift between consecutive days for cells classified as retrospective, present and prospective grid cells on the preceding day, together with shuffled controls generated by randomly pairing cells across days (Kruskal-Wallis tests with p-values indicated). **(k)** Relationship between the absolute change in optimal shift across consecutive days and the maximum gridness score on the subsequent day, pooled across the three grid cell classes. Red line indicates the linear regression fit across cells (Pearson correlation coefficient and p-value indicated).

**Supplementary Figure 3. Example retrospective grid coding in mouse medial entorhinal cortex.**

Fifteen example grid cells with maximum gridness score with backward shifted position. (Left) Spatial rate maps (top, color coded), event maps (center, path in gray and events in black dots) and auto-correlograms (bottom, color coded) at original and backward shifted positions. (Right top) Gridness scores by the shifted positions, where positive and negative shifts indicate forward and backward projections, respectively. Red dashed line, threshold of gridness score to identify grid cells; gray dashed line, original position. (Right bottom) Head direction tuning curves (black) together with directional behavior (gray) at the original position. MVL, mean vector length; peak, peak event rate in Hz.

**Supplementary Figure 4. Electrode recording reveal retrospective and prospective grid coding in mouse entorhinal cortex.**

**(a)** Representative firing patterns of three prospective grid cells. (Left) Spatial rate maps (top, color coded), event maps (center, path in gray and events in black dots) and auto-correlograms (bottom, color coded) at original and forward shifted positions. (Right top) Gridness scores by the shifted positions, where positive and negative shifts indicate forward and backward projections, respectively. Red dashed line, threshold of gridness score to identify grid cells; gray dashed line, original position. (Right bottom) Head direction tuning curves (black) together with directional behavior (gray) at the original position. MVL, mean vector length; peak, peak event rate in Hz. **(b)** Same as (a) but for six representative retrospective grid cells. **(c)** (Top) Gridness scores of all retrospective (left) and prospective (right) grid cells by shifted positions (color coded, n = 228 and 274 cells from 18 animals). Black line, original position. (Bottom) Distribution of retrospective and prospective grid cells by shifted positions across animals (color coded). Red dashed line, average optimal position shift of all retrospective and prospective grid cells, respectively. **(d)** From left to right: proportion, grid spacing, position shift and optimal position shift relative to grid spacing of all retrospective, present and prospective grid cells (Friedman test across animals, the fraction of cells: S = 9.9, p = 0.006, spacing: S = 0.2, p = 0.9; Kruskal-Wallis test across cells, shift: H = 593, p = 10^-128^, normalized shift: H = 382, p = 10^-83^). The data were obtained from both our electrophysiological recordings and the publicly available dataset.

**Supplementary Figure 5. Comparison of grid cell coding properties across datasets.**

Proportions and optimal position shifts normalized by grid spacing are shown for retrospective, present and prospective grid cells identified in our tetrode recordings and in the publicly available dataset (Kruskal-Wallis tests with p-values indicated).

**Supplementary Figure 6. Retrospective grid coding maintains across days.**

Representative firing patterns of seven retrospective grid cells across three days, and of two retrospective grid cells across two days. All recordings were obtained from repeated sessions conducted in the same familiar environment using our electrophysiological dataset.

**Supplementary Figure 7. General linearized model confirms retrospective grid cells encode past locations.**

**(a)** Schematic of general linearized model (GLM) framework. Positions are first projected onto Gaussian basis functions and then passed through an exponential nonlinearity to compute the mean rate of a Poisson process. The filled bin denotes the animal’s current location. **(b)** Observed (top, color coded) and GLM predicted (bottom) spatial rate maps of six representative cells. **(c)** Gridness score, explained deviance and spatial information at forward-shifted positions in two representative prospective grid cells. (Left) Spatial rate maps (top, color coded), event maps (center, path in gray and events in black dots) and auto-correlograms (bottom, color coded) at original and forward shifted positions. (Middle top) Gridness scores by the shifted positions, where positive and negative shifts indicate forward and backward projections, respectively. Red dashed line, threshold of gridness score to identify grid cells; gray dashed line, original position. (Middle bottom) Head direction tuning curves (black) together with directional behavior (gray) at the original position. MVL, mean vector length; peak, peak event rate in Hz. (Right top) Explained deviance of GLM across spatial shifts. The Pearson correlation coefficient with gridness scores across spatial shifts is indicated. (Right bottom) Spatial information across different spatial shifts. **(d)** Same as (c), but for two representative retrospective grid cells. **(e)** For all retrospective and prospective grid cells, the distribution of Pearson correlation between gridness scores and explained deviance of GLM across spatial shifts, compared with shuffled data (Kruskal-Wallis test across cells with p-values indicated; n = 228 and 274 for retrospective and prospective grid cells, respectively). **(f)** Same as (e), but for the Pearson correlation between gridness scores and spatial information.

**Supplementary Figure 8. Retrospective grid coding in rat entorhinal cortex is phase-locked.**

**(a)** Representative firing patterns of three prospective grid cells. (Left) Spatial rate maps (top, color coded), event maps (center, path in gray and events in black dots) and auto-correlograms (bottom, color coded) at original and forward shifted positions. (Right top) Gridness scores by the shifted positions, where positive and negative shifts indicate forward and backward projections, respectively. Red dashed line, threshold of gridness score to identify grid cells; gray dashed line, original position. (Right bottom) Distribution of theta phase at which each spike occurred. **(b)** Same as (a) but for six representative retrospective grid cells. **(c)** (Top) Gridness scores of all retrospective (left) and prospective (right) grid cells by shifted positions (color coded, n = 178 and 120 cells from 4 animals). Black line, original position. (Bottom) Distribution of retrospective and prospective grid cells by shifted positions across animals (color coded). Red dashed line, average optimal position shift of all retrospective and prospective grid cells, respectively. **(d)** Distribution across all retrospective (top) and prospective (bottom) grid cells of the average theta phase preferences. Retrospective grid cells are clustered into two groups based on preferred theta phase. **(e)** Distribution of proportion and optimal position shift relative to grid spacing of all retrospective, present and prospective grid cells, and for two groups of retrospective grid cells (fraction of cells across animals: Friedman test for between-group comparison, Wilcoxon test for retrospective groups; normalized shift: Kruskal-Wallis test for between-group comparison, Mann-Whitney test for retrospective groups). Retrospective grid cells were separated into two subgroups based on their preferred theta phase, corresponding to the two clusters shown in (d). The data were obtained from the publicly available dataset.

**Supplementary Figure 9. Comparison of spatial and temporal shifts in retrospective coding.**

**(a)** Example firing patterns and gridness scores of a retrospective grid cell under spatial (left) and temporal (right) shifts. (Left) Spatial rate maps (top, color coded), event maps (center, path in gray and events in black dots) and auto-correlograms (bottom, color coded) at original or backward shifted positions. (Right) Gridness scores by the shifted positions or time, where positive and negative shifts indicate forward and backward projections, respectively. Red dashed line, threshold of gridness score to identify grid cells; gray dashed line, original position or time. **(b)** (Top) Gridness scores of all retrospective (left) and prospective (right) grid cells by shifted time (color coded, n = 861 and 1,335 cells from 8 animals). Black line, original time. (Bottom) Distribution of retrospective and prospective grid cells by shifted time across 8 animals (color coded). Red dashed line, average optimal temporal shift of all retrospective and prospective grid cells, respectively. **(c)** Distribution of gridness score and spatial information at the optimal position and temporal shifts across all retrospective and prospective grid cells identified based on position shifts (n = 959 and 1,465 cells, respectively; two-sided Wilcoxon signed rank tests with p-values indicated).

**Supplementary Figure 10. Retrospective grid coding varies with sensory cues.**

**(a)** Spatial coverage in ENV1, ENV2 and ENV3 (n = 3 animals per environment, Mann-Whitney tests with p-values indicated). Only the first 30 minutes of each ENV1 and ENV2 session were analyzed to match the duration of ENV3 sessions. **(b)** Distribution of relative grid shift among retrospective (left) and prospective (right) grid cells across the three environments (ENV1, ENV2 and ENV3 contained 257, 505 and 197 retrospective cells and 644, 591 and 230 prospective cells, respectively; Kruskal-Wallis tests across cells, retrospective: H = 3.5, p = 0.17; prospective: H = 90.45, p = 10^-19^). **(c)** Changes in gridness score (left) and spatial information (right) between light and dark conditions for retrospective, present and prospective grid cells (n = 112 retrospective, 34 present and 137 prospective cells, respectively; Kruskal-Wallis test across cells with p-values indicated). For each condition, both metrics were calculated at the optimal shift independently identified under that condition. **(d)** Pearson correlation coefficient between retrospective cell-pair correlation matrices obtained during light and dark sessions (red line, observed value indicated), together with the distribution obtained after shuffling cell identities (gray, one-tailed permutation test with p-value indicated).

**Supplementary Figure 11. Sensory cues are required to maintain prospective grid coding.**

**(a)** Representative firing patterns of two prospective grid cells under light and dark conditions. Spatial rate maps (row 1, color coded), event maps (row 2, path in gray and events in black dots) and auto-correlograms (row 3, color coded) at original and forward shifted positions. Row 4 shows gridness scores by the shifted positions, where positive and negative shifts indicate forward and backward projections, respectively. Red dashed line, threshold of gridness score to identify grid cells; gray dashed line, original position. **(b)** Distribution of gridness scores (left) and spatial information (right) across all prospective grid cells (n = 137 cells, Kruskal-Wallis test with p-values indicated). For “Light” and “Dark”, metrics were computed using the optimal shift identified during light condition; for “Dark (opt. shift)”, the optimal shift was determined separately from the dark condition. **(c)** Distributions across prospective grid cells of optimal position shifts under light and dark conditions (two-tailed Wilcoxon matched-pairs signed rank tests p-values indicated). **(d)** For each prospective grid cell pair, the Pearson correlation between their calcium activity traces under light and dark conditions. Red line indicates the linear regression across cell-pair correlations (Pearson correlation coefficient and p-value indicated).

**Supplementary Figure 12. Sensory cues are required to maintain grid coding.**

**(a)** Representative examples of trajectories during light (left) and dark (right) conditions. **(b)** Distribution across sessions of the spatial coverage and speeds under light and dark conditions (n = 5 sessions, two-tailed Wilcoxon matched-pairs signed rank tests p-values indicated). **(c)** Firing patterns of four representative present grid cells under light and dark conditions. Each group of subpanels shows the spatial rate map (first row; color coded), the event map (second row; trajectory in gray and events in black dots) and the corresponding auto-correlogram (third row; color coded). **(d)** Distribution across present grid cells of gridness scores (left) and spatial information (right) under light and dark conditions (n = 34 cells, two-tailed Wilcoxon matched-pairs signed rank tests p-values indicated). **(e)** For each present grid cell pair, the Pearson correlation between their calcium activity traces under light and dark conditions. Red line indicates the linear regression across cell-pair correlations (Pearson correlation coefficient and p-value indicated).

**Supplementary Figure 13. Sensory cues are required to maintain retrospective and prospective grid coding for each session.**

**(a)** Retrospective grid coding is disrupted in darkness while network structure is preserved across sessions. (i) Distribution across sessions of gridness scores (left) and spatial information (right) (n = 5 sessions, Mann-Whitney test with p-values indicated). For “Light” and “Dark”, metrics were computed using the optimal shift identified during light condition; for “Dark (opt. shift)”, the optimal shift was determined separately from the dark condition. (ii) Pearson correlation of calcium activity traces between all retrospective grid cell pairs under light and dark conditions. Lines indicate the linear regression across cell-pair correlations for each session (color-coded by session). Inset: Distribution of Pearson correlation coefficients across sessions. **(b)** Same as (a) but for prospective grid cells.

**Supplementary Figure 14.** **Loss of self-motion input impairs present grid coding.**

**(a)** Representative examples of trajectories under free exploration (left) and assisted navigation (right). **(b)** Distribution across sessions of spatial coverage and average speeds for each condition (n = 5 sessions, two-tailed Wilcoxon matched-pairs signed rank tests p-values indicated). **(c)** Distribution of Pearson correlation coefficients among retrospective grid-cell pairs during free and assisted movement, together with the shuffled controls (Kruskal-Wallis tests with p-values indicated). **(d)** Pearson correlation between the corresponding pairwise correlation coefficients obtained during free and assisted movement (red line, observed value indicated). Gray distribution, null distribution generated by shuffling cell identities (one-sided permutation test with p-value indicated). **(e)** Firing patterns of three representative present grid cells. Each group of subpanels shows the spatial rate map (first row; color coded), the event map (second row; trajectory in gray and events in black dots; Peak: peak event rate in Hz), the corresponding auto-correlogram (third row; color coded) and the head direction map (fourth row; behavioral map in gray and rate map in black; MVL, mean vector length) across sessions (columns). **(f)** Distribution across present grid cells of gridness scores, spatial information and MVL under free and assisted conditions (n = 40 cells, two-tailed Wilcoxon matched-pairs signed rank tests p-values indicated). **(g)** For each present grid cell pair, the Pearson correlation between their calcium activity traces under free and assisted conditions. Red line indicates the linear regression across cell-pair correlations (Pearson correlation coefficient and p-value indicated).

**Supplementary Figure 15. Loss of self-motion input impairs prospective grid coding.**

**(a)** Example firing patterns of two prospective grid cells during free exploration and assisted movement. (Left) Spatial rate maps (top, color coded), event maps (center, path in gray and events in black dots) and auto-correlograms (bottom, color coded) at original and forward shifted positions. (Right top) Gridness scores by the shifted positions, where positive and negative shifts indicate forward and backward projections, respectively. Red dashed line, threshold of gridness score to identify grid cells; gray dashed line, original position. (Right bottom) Head direction tuning curves (black) together with directional behavior (gray) at the original position. MVL, mean vector length; peak, peak event rate in Hz. **(b)** Distribution of gridness score (left), spatial information (middle) and MVL (right) across all prospective grid cells (n = 204 cells, Kruskal-Wallis test with p-values indicated). For “Free” and “Assisted”, metrics were computed using the optimal shift identified during free foraging; for “Assisted (opt. shift)”, the optimal shift was determined separately from the assisted session. **(c)** For each prospective grid cell pair, the Pearson correlation between their calcium activity traces under free and assisted conditions. Red line indicates the linear regression across cell-pair correlations (Pearson correlation coefficient and p-value indicated).

**Supplementary Figure 16. Loss of self-motion input impairs both retrospective and prospective grid coding for each session.**

**(a)** Retrospective grid coding and network structure is disrupted during assisted movement across sessions. (i) Distribution across sessions of gridness scores (left), spatial information (middle) and MVL (right) (n = 5 sessions, Mann-Whitney test with p-values indicated). For “Free” and “Assisted”, metrics were computed using the optimal shift identified during free foraging; for “Assisted (opt. shift)”, the optimal shift was determined separately from the assisted session. (ii) Pearson correlation of calcium activity traces between all retrospective grid cell pairs under free and assisted conditions. Lines indicate the linear regression across cell-pair correlations for each session (color-coded by session). Inset: Distribution of Pearson correlation coefficients across sessions. **(b)** Same as (a) but for prospective grid cells.
